# Research on Straightness Perception Compensation Model of FBG Scraper Conveyor Based on Rotation Error Angle

**DOI:** 10.3390/s22176399

**Published:** 2022-08-25

**Authors:** Yang Song, Xinqiu Fang, Gang Wu, Ningning Chen, Minfu Liang, Ziyue Xu, Fan Zhang

**Affiliations:** 1 School of Mines, China University of Mining and Technology, Xuzhou 221116, China; 2 Research Center of Intelligent Mining, China University of Mining and Technology, Xuzhou 221116, China

**Keywords:** straightness perception, scraper conveyor, accuracy compensation model, finite element simulation, FBG curvature sensor

## Abstract

The accurate perception of straightness of a scraper conveyor is important for the construction of intelligent working faces in coal mines. In this paper, we propose a precision compensation model based on rotation error angle to improve the accuracy of the fiber Bragg grating (FBG) curvature sensor of a scraper conveyor. The correctness of the model is verified by theoretical analysis, numerical simulation, and experiments. Finally, the feasibility of the model is analyzed and discussed for field application in a coal mine. When the rotation error angle is within the range of 0~90°, according to the strain of FBG obtained by numerical simulation, the radius of the curvature is inversely calculated by the compensation model. The relative error of each discrete point is within ±0.9%, and the relative error after fitting is less than 0.2%. The experiment shows that the relative error of the curvature radius after fitting according to the theoretical formula is less than ±3%, and the relative error of the curvature radius value obtained by the inverse deduction of each discrete point is less than ±6%, which verifies the correctness and applicability of the compensation model. In addition, the compensation model with the FBG curvature sensor has broad application prospects in coal mine underground conveyors, submarine pipelines and ground pipelines.

## 1. Introduction

As one of the key technologies in the construction of intelligent working faces, the straightness perception of a scraper conveyor is of great significance to the realization of “three straights and two levels” of the working face; that is, the straightness of a scraper conveyor can provide important data support for the adaptive straightening of hydraulic support, the self-straightening of the scraper conveyor, the straight cutting of the shearer, and the flatness of the roof and floor of the working face [1,2,3]. Therefore, the realization of high-precision straightness sensing of scraper conveyors is of great significance for the construction of intelligent working faces.

In order to realize the perception of straightness of scraper conveyors, scholars have explored technologies including visual measurement, position sensing, inertial navigation system and so on. Yang Zhu [4] proposed the image acquisition and processing theory of a complex working face based on visual measurement technology to realize straightness measurements in view of the problems of dark light and coal dust faced by visual measurement technology in the working face. Liu Pengkun et al. [5] arranged structural light along the scraper conveyor, installed cameras under the top beam of the hydraulic support, and finally used the visual algorithm to measure the shape of the scraper conveyor in real time. Yu Jiaxin et al. [6] and Niu Jianfeng et al. [7] both proposed to install a position sensor on the hydraulic support so as to obtain the relative position relationship between the hydraulic support and the coal wall or the scraper conveyor, and finally obtain the straightness of the scraper conveyor based on the hydraulic support. The landmark project carried out by the Commonwealth Scientific and Industrial Research Organization (CSIRO) of Australia proposed the idea of retrieving the shape of the scraper conveyor by detecting the three-dimensional path of the shearer through inertial navigation technology [8,9,10]. However, with the above sensing methods, it is difficult to execute real-time monitoring of the straightness of the scraper conveyor for a long time. Secondly, the above scraper conveyor straightness sensing methods all use electromagnetic sensors, which are vulnerable to adverse environmental factors such as coal dust, water mist and strong electromagnetic on the working face, resulting in a significant decline in the sensing accuracy after a long period of work.

With the wide application of various FBG sensors [11,12,13,14,15] in coal mines and other engineering fields, Wang Guofa et al. [16] and Ge Shirong et al. [17] believe that the FBG sensing method provides a reliable means for intelligent information sensing and reliable transmission in coal mines. In addition, FBG shape sensing technology has long been developed and applied in aviation [18], robotics [19], pipeline monitoring [20], and medical fields [21]. In view of this, Fang Xinqiu et al. [22] developed the FBG curvature sensor of the scraper conveyor based on the intelligent sensing of coal mines, and preliminarily solved the above problems by applying the FBG shape sensing technology to the straightness sensing of the scraper conveyor. However, the sensor will rotate after long-term operation, resulting in angular deviation from the theoretical installation position and large errors in the process of curvature inversion.

Based on the application of the FBG curvature sensor [22] of a scraper conveyor, a precision compensation model based on rotation error angle is proposed in this paper. The feasibility of the model is verified by theoretical analysis, simulation, and experiments. This research is significant for the real-time online high-precision sensing of the straightness of the scraper conveyor in the working face. The accuracy compensation model based on the rotation error angle proposed in this paper has considerable engineering application value and application prospects with the use of the FBG curvature sensor of a scraper conveyor.

## 2. Sensor Theory Analysis and Compensation Model Establishment

### 2.1. Basic Sensing Theory of FBG

**Figure 1:**
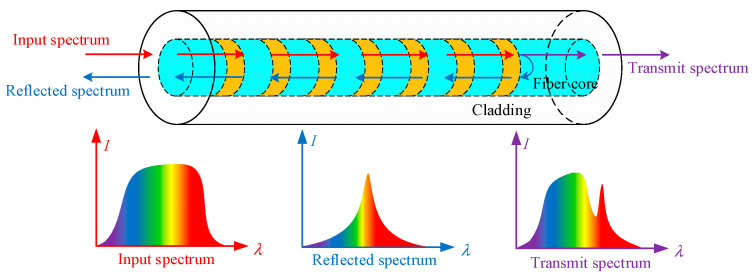
Schematic diagram of FBG sensing.

FBG is made by exposing the core of a single-mode fiber to strong ultraviolet light with periodic patterns. The refractive index of the fiber core thus changes periodically, resulting in regular changes in the transmission spectrum and reflection spectrum of light waves when they are transmitted in the fiber grating. The essence of its role is to form a narrow-band filter in the fiber core [23,24]. The sensing principle of FBG is shown in Figure 1.

When a beam of light passes through the FBG, the spectrum meeting the Bragg grating conditions will be reflected, and the peak of the reflected spectrum is the FBG wavelength. The Bragg wavelength is determined by the effective refractive index of the fiber core and the grating period [25,26], and its basic expression is:

(1) $$\lambda_{B}=2n_{eff}\Lambda ,$$

where *λ_B_* is the peak wavelength of the reflection spectrum, *n_eff_* is the effective refractive index of the core, and Λ is the grating period.

FBG has the characteristics of temperature strain cross-sensitivity. The thermo-optic effect and thermal expansion effect caused by temperature change will cause changes in the effective refractive index and grid spacing of FBG. Strain will cause a change in the grating period, and the elasto-optic effect will also cause a change in the effective refractive index of FBG. Therefore, the relationship between FBG wavelength change and temperature and strain can be expressed as [27]:

(2) $$\frac{\Delta \lambda_{B}}{\lambda_{B}}=\left(1-p_{e}\right)\varepsilon_{z}+\left(\alpha +\xi \right)\Delta T,$$

where Δ*λ* is the wavelength variation of FBG, *p_e_* is the effective elastic optic coefficient, *α* and *ξ* are the thermal expansion coefficient and thermal optical coefficient of optical fiber, *ε_z_* is the axial strain of the FBG, and Δ*T* is the temperature change.

According to the coal mine safety regulations, the working face temperature should be less than 26 °C. Moreover, the working face temperature of the modern intelligent mine is at the optimal temperature suitable for the human body, which is regarded as a constant temperature environment, so the influence of temperature on FBG is ignored. Under constant temperature, FBG is only affected by strain, and the wavelength change of its reflected light is positively correlated with the axial stress of the fiber [28]:

(3) $$\Delta \lambda_{B}=(1-p_{e})\varepsilon_{z}\lambda_{B},$$

### 2.2. Straightness Sensing Principle of Scraper Conveyor Based on FBG

The straightness perception of a scraper conveyor based on FBG is to sense its shape by arranging FBG curvature sensors along the scraper conveyor. After the scraper conveyor is moved by the hydraulic support to produce curvature changes, it will transmit its own curvature changes to the FBG curvature sensor at the same time. According to the relationship between the wavelength change of the FBG curvature sensor and the curvature, the morphological changes of the scraper conveyor can be deduced.

**Figure 2:**
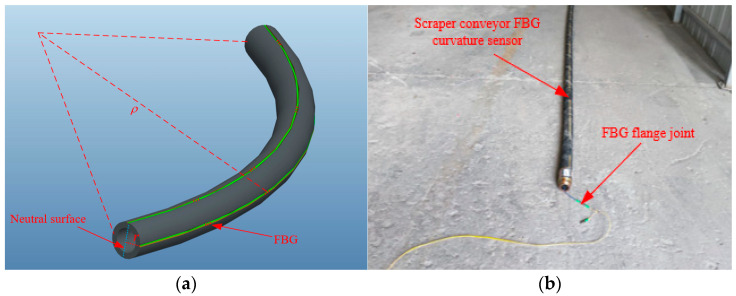
Schematic diagram and photo of FBG curvature sensor of scraper conveyor. (**a**) Schematic diagram; (**b**) photo.

The FBG curvature sensor involved in this study is based on the orthogonal arrangement of FBG strings on the circular cross-section elastic substrate, as shown in Figure 2. When pure bending occurs to the sensor, the strain transfer efficiency is ignored, and the axial strain and curvature of FBG attached to the sensor substrate have the following relationship [22]:

(4) $$\varepsilon_{z}=\frac{r}{\rho }=rK,$$

where *r* is the distance from the fixed surface of the FBG to the neutral surface, *ρ* is the curvature radius of the measuring point, and *K* is the curvature corresponding to the point.

Equation (3) shows the relationship between the wavelength change of FBG and its axial strain; Equation (4) shows the relationship between the curvature of the measuring point and the axial strain of the FBG. According to Equations (3) and (4), the relationship between the curvature of the measuring point and the wavelength change of the FBG is:

(5) $$K=\frac{\Delta \lambda_{B}}{(1-P_{\varepsilon })\lambda_{B}r},$$

In Equation (5), $(1-P_{\varepsilon })\lambda_{B}r=M$, where *M* is the curvature sensitivity coefficient of FBG, which is a quantity related to FBG and the sensor substrate. When the sensor is manufactured and works according to the theoretical conditions, *M* is a fixed value. Therefore, Equation (5) can be simplified as:

(6) $$\Delta \lambda_{B}=KM$$

### 2.3. Construction of Accuracy Compensation Model Based on Rotation Error Angle

**Figure 3:**
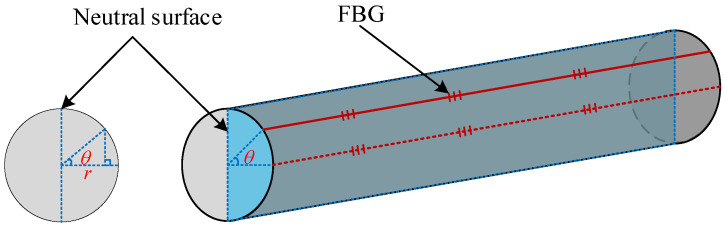
Schematic diagram of the FBG curvature sensor generating a rotation error angle.

As shown in Figure 3, the included angle between the actual position of FBG and the theoretical position of FBG is defined as the rotation error angle. When the FBG curvature sensor of scraper conveyor is not installed according to the installation standard or after long-term operation, it will produce a rotation error angle. When the rotation angle error occurs, according to the geometric relationship, the relevant parameters of FBG become:

(7) $$\left\{\begin{array}{l} \rho^{\prime }=\rho -r\left(1-\cos\theta \right)\\ K^{\prime }=\frac{1}{\rho -r\left(1-\cos\theta \right)}\\ r^{\prime }=r\cos\theta \end{array}\right.,$$

where *ρ*′ is the radius of the curvature when the FBG is in the actual position, *K*′ is the curvature when the FBG is in the actual position, *r*′ is the distance from the neutral surface when the FBG is in the actual position, and *θ* is the rotation error angle.

Affected by the rotation error angle, the strain of FBG at the actual position will also change. It can be seen from Equations (4) and (7):

(8) $$\varepsilon =\frac{r\cos\theta }{\rho -r\left(1-\cos\theta \right)}$$

In addition, the curvature of FBG in the actual position will change compared with that in the theoretical position. According to the mathematical relationship between the radius of the curvature and the curvature and Equation (7), the relationship between the curvature of FBG in the theoretical position and its curvature in the actual position is:

(9) $$K=\frac{K^{\prime }}{1+rK^{\prime }\left(1-\cos\theta \right)},$$

where *K*′ is the curvature of FBG in the actual position.

According to Equations (6) and (7), after the rotation error angle is generated, the following relationship exists between the wavelength change of FBG and the rotation error angle:

(10) $$\Delta \lambda =(1-P_{\varepsilon })\lambda_{B}r\cos\theta K^{\prime }$$

Equations (9) and (10) can establish the FBG curvature perception accuracy compensation formula based on the rotation error angle:

(11) $$K=\frac{\Delta \lambda }{(1-P_{\varepsilon })\lambda_{B}r\cos\theta +\left(1-\cos\theta \right)\Delta \lambda r}$$

Substituting the curvature sensitivity coefficient of FBG into Equation (11) can be simplified as:

(12) $$K=\frac{\Delta \lambda }{M\cos\theta +\left(1-\cos\theta \right)\Delta \lambda r}$$

From Equation (12), the curvature of FBG in the theoretical position can be obtained by the wavelength change of FBG in the actual position. That is, when the FBG deviates from the preset theoretical position due to the rotation of the sensing substrate, the accuracy of its curvature measurement can be compensated by substituting the rotation angle into the above equation.

## 3. Finite Element Simulation

In this numerical simulation, ANSYS Workbench software was used to analyze the strain characteristics of FBG under different rotation error angles of the FBG curvature sensor used in the scraper conveyor. Through the simulation results, according to the accuracy compensation model based on the rotation error angle, the curvature radius was calculated to verify the effectiveness and rationality of the accuracy compensation model.

**Figure 4:**
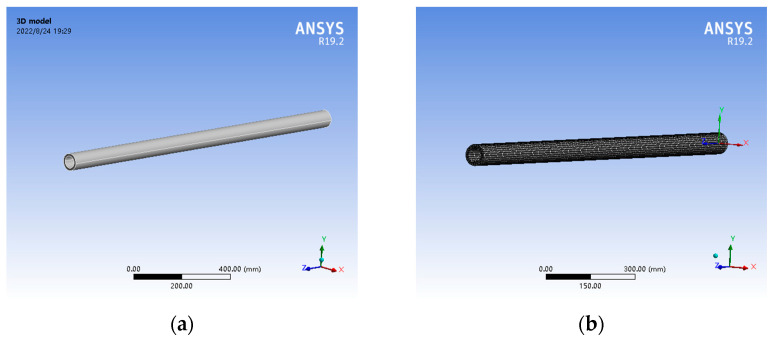
Three-dimensional solid model and finite element calculation model of FBG curvature sensor. (**a**) Three-dimensional solid model; (**b**) finite element calculation model.

The FBG curvature sensor of the scraper conveyor has an inner diameter of 51 mm, an outer diameter of 72 mm and a simulated length of 1500 mm. According to the experimental materials, the elastic modulus of the matrix material is set as 6.11 mpa and Poisson’s ratio is set as 0.49. Based on this, a three-dimensional solid model is established and meshed to generate a finite element analysis model. The total number of elements in the model is 9964 and the number of nodes is 53,033, as shown in Figure 4.

By imposing a fixed constraint on the negative end of the Z-axis of the sensor model and a negative bending moment around the X-axis on the positive end of the Z-axis, the bending of the sensor in the YOZ plane is simulated, and the bending curvature of the sensor can be obtained by deriving the node displacement of the sensor and fitting the curve. Therefore, the loading can be adjusted many times by dichotomy to control the radius of the curvature of this simulation to 15,000 mm, and then the orthogonal FBG strain data can be measured by inserting a path under different rotation error angles of the sensor model.

**Figure 5:**
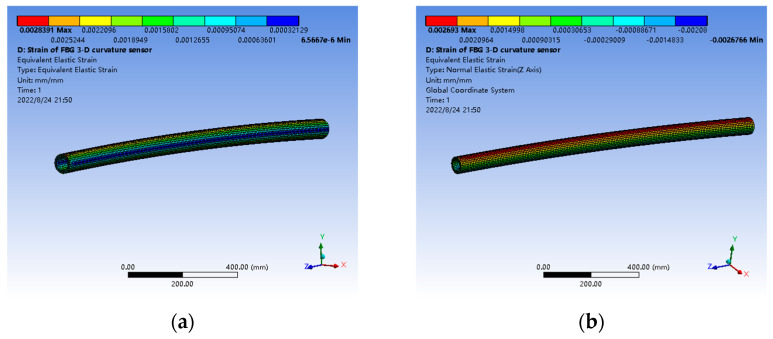
Nephogram of equivalent strain and Z-direction elastic strain of FBG curvature sensor when the curvature radius is 15,000 mm. (**a**) Equivalent strain; (**b**) Z-direction elastic strain.

When the FBG curvature sensor bends with a curvature radius of 15,000 mm, its equivalent strain and Z-direction elastic strain nephogram are as in Figure 5. It can be seen from Figure 5a that the FBG curvature sensor has negative bending along the Y-axis. After bending, the equivalent strain on the surface of the sensor macroscopically presents the characteristics of symmetrical distribution along the neutral surface. The surface strain of the neutral surface after bending is the smallest. With the transition to both sides of the neutral surface, the surface strain value gradually increases. In the plane where bending occurs, the innermost and outermost surface strains of the sensor are the largest. It can be seen from Figure 5b that when the FBG curvature sensor bends as shown in the figure, the strain on the neutral surface of the sensor is almost zero. On the positive side of the neutral surface along the Y-axis, the Z-direction elastic strain of the sensor is positive, that is, tensile strain. On the negative side of the neutral surface along the Y-axis, the Z-direction elastic strain of the sensor is negative, that is, the compressive strain.

**Figure 6:**
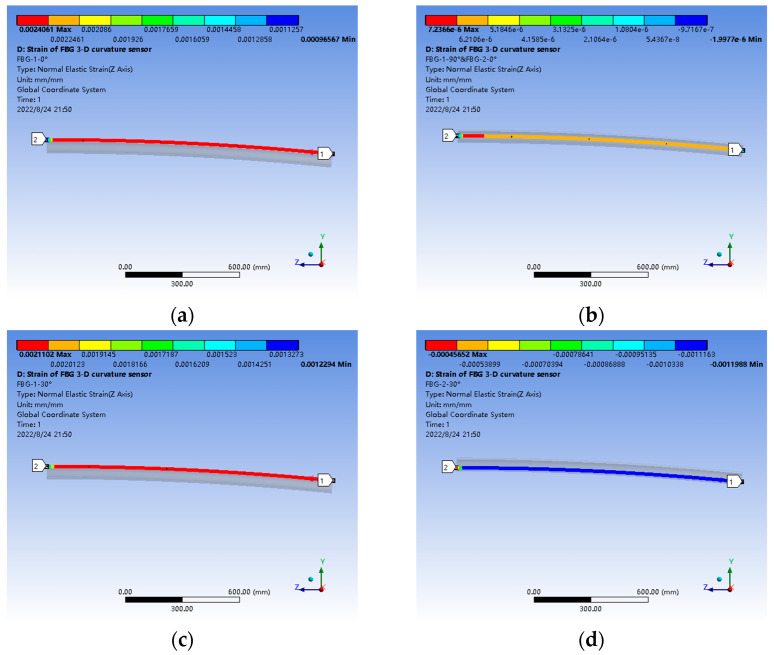

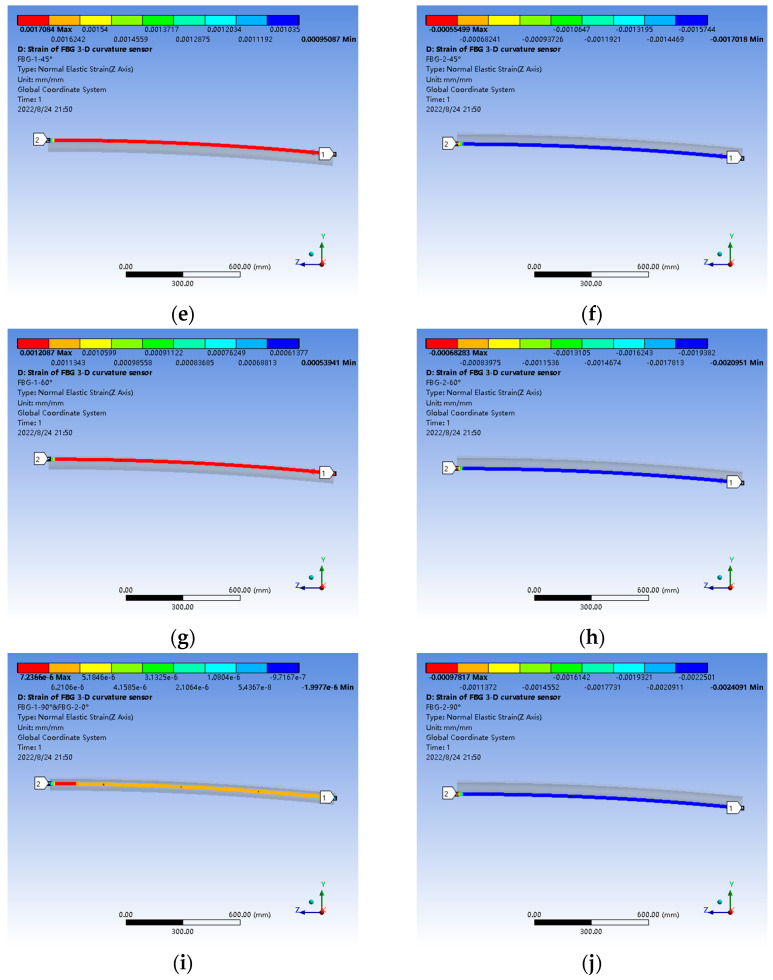
Strain nephogram of orthogonal FBG under different rotation error angles. (**a**) The rotation angle error of FBG I is 0°; (**b**) the rotation angle error of FBG II is 0°; (**c**) the rotation angle error of FBG I is 30°; (**d**) the rotation angle error of FBG II is 30°; (**e**) the rotation angle error of FBG I is 45°; (**f**) the rotation angle error of FBG II is 45°; (**g**) the rotation angle error of FBG I is 60°; (**h**) the rotation angle error of FBG II is 60°; (**i**) the rotation angle error of FBG I is 90°; (**j**) the rotation angle error of FBG II is 90°.

Figure 6 shows the strain distribution nephogram of FBG under different rotation error angles after the FBG curvature sensor with an orthogonal arrangement bends with a curvature radius of 15,000 mm along the negative direction of the Y-axis. It can be seen from the figure that under the influence of the rotation error angle, the FBG strain of the orthogonal arrangement will produce errors compared with the FBG strain when the rotation error angle is zero. Secondly, as the rotation error angle gradually increases from 0° to 90°, the strain of FBG I in the two grating strings arranged orthogonally will gradually decrease, and the strain of FBG II will gradually increase.

It can be seen that since the FBG curvature sensor maintains a specific curvature of 15,000 mm in the simulation, its surface strain is roughly equal along the axis direction, and the surface strain value shows a gradual change and symmetrical distribution along the circumference, ignoring the stress concentration at the two ends of the constraint and load. In the rotation error angle range of 0~90°, FBG I is in tension, and its strain value decreases with the increase in rotation error angle. In the rotation error angle range of 0~90°, FBG II is compressed, and its strain value increases with the increase in rotation error angle.

Ignoring the strain transfer efficiency between the FBG and the matrix material, the path center strain value is derived in ANSYS Workbench, which can be regarded as the strain value of the FBG. Based on the simulated strain value, the curvature radius can be deduced by adopting the compensation model based on the rotation angle error, and the respective relative errors can be calculated, as shown in Table 1. After calculation, the relative error between the simulated strain value and the theoretical strain value is within ±0.6%, which shows the correctness of the theory adopted in the simulation. The relative errors of the curvature radius calculated based on the theoretical model are all within ±0.9%, which verifies the compensation model based on the rotation angle error.

Substituting the parameters of this simulation setting into Equation (8), it can be seen that the strains of FBG I and FBG II can be expressed as:

(13) $$\left\{\begin{array}{l} \varepsilon_{I}=\frac{36\times \cos\theta }{15000-36\times \left(1-\cos\theta \right)}\\ \varepsilon_{\mathrm{II}}=\frac{36\times \sin\theta }{36\times \left(1-\sin\theta \right)-15000} \end{array}\right.,$$

**Figure 7:**
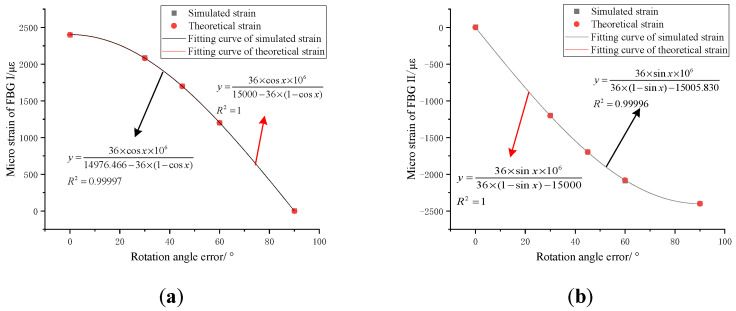
Relationship between FBG strain and rotation error angle in an orthogonal arrangement. (**a**) Micro strain fitting curve of FBG I; (**b**) micro strain fitting curve of FBG II.

The scatter diagram is drawn based on the data in Table 1. When the relevant parameters of the sensor substrate are fixed, it is fitted according to the user-defined theoretical formula, as shown in Figure 7. It can be seen from Figure 7a that when the parameter *r* is fixed, the curvature radius parameter in the fitting formula is 14,976.466 mm, and the relative error is 0.157%. It has a high fitting degree of 0.99997, and the fitting curve conforms to the theoretical analysis. When the rotation error angle is within 0–90°, FBG I is in tension, its strain value gradually decreases and its reduction rate gradually increases. It can be seen from Figure 7b that when the parameter *r* is fixed, the curvature radius parameter in the fitting formula is 15,005.830 mm, the relative error is 0.039%, and its fitting degree is 0.99996, which is also a high fitting degree. This fitting curve conforms to the theoretical analysis. When the rotation error angle is within 0–90°, FBG II is compressed, its strain value gradually increases and its increasing rate gradually decreases. Through the fitting analysis of discrete points of FBG I and FBG II, the relative errors are less than 0.2%, which further verifies the compensation model based on the rotation angle error.

## 4. Experiment and Result Analysis

### 4.1. Experimental System

**Figure 8:**
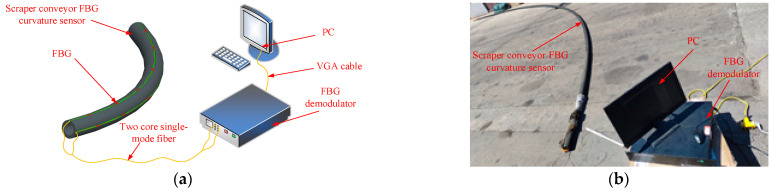
Curvature rotation error angle test system of FBG curvature sensor of scraper conveyor. (**a**) Experimental schematic diagram; (**b**) photo of experiment process.

The curvature–rotation error angle test system of the FBG curvature sensor of the scraper conveyor is shown in Figure 8. In the experimental preparation stage, we first marked an arc with a curvature radius of 15,000 mm on the test site, and marked the angle on both ends of the sensor substrate. In the experiment, the FBG curvature sensor was placed along the marked arc to control the curvature, and the rotation error angle was controlled according to the angle marks on the two end faces of the sensor substrate. The FBG curvature sensor is connected to the FBG demodulator through an optical fiber and FC/APC connector, and the demodulator is connected to the display screen.

HKA-M FBG demodulator is mainly used for data acquisition. The demodulator is integrated with the computer and equipped with data demodulation software. Its wavelength range is 1525~1565 nm and wavelength resolution is 1 pm. The grating used in the sensor is directly written into the single-mode fiber in the core by ultraviolet laser. The length of the grid area is 10mm, and the initial center wavelength of the FBG curvature sensor of the scraper conveyor after packaging is shown in Table 2.

### 4.2. Experimental Results and Analysis

**Figure 9:**
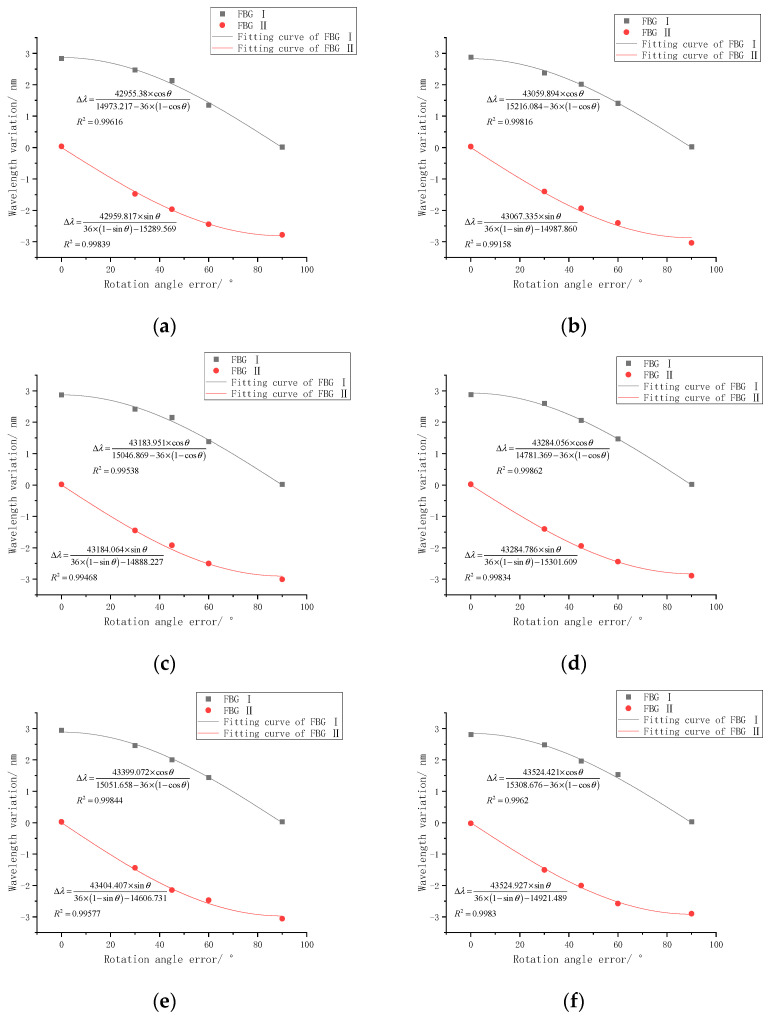
Variation curve of FBG wavelength with rotation error angle (fixed parameters *r* and *M*, fitting parameters *ρ*). (**a**) Grating 1; (**b**) grating 2; (**c**) grating 3; (**d**) grating 4; (**e**) grating 5; (**f**) grating 6.

When the curvature radius of the FBG curvature sensor of the scraper conveyor is kept at 15,000 mm, and the rotation error angle is changed, the reflection center wavelength of each FBG of the sensor will shift. According to the statistical data of the rotation error angle of the sensor’s field work, the rotation error angle was controlled at 0~90° in the experiment. The variations of the wavelength of each grating area of FBG I and FBG II with the rotation error angle in the experiment are shown in Figure 9.

It can be seen from the data in Figure 9 and the user-defined fitting formula that the fitting coefficient between the reflection wavelength data of each FBG and the rotation error angle fitting formula and the theoretical formula is higher, both above 0.99, indicating that the mathematical relationship between the wavelength change of FBG and the rotation error angle conforms to the theoretical derivation. According to the fitting formula in Figure 9, the curvature radius obtained by fitting is shown in Table 3. It can be seen from Table 3 that when the parameters *r* and *M* are fixed, the relative errors of the parameters *ρ* are within 3%, which also verifies the correctness of the fitting formula.

According to the variation of the FBG central wavelength measured in the experiment, combined with the parameters such as rotation angle error, the curvature value of the FBG curvature sensor of the scraper conveyor is deduced through the accuracy compensation model based on the rotation error angle, as shown in Table 4. Compared with the calibration curvature value, the curvature value calculated by the theoretical compensation model is close to the calibration value, and the relative error is within 6%, which confirms the correctness of the accuracy compensation model. There is a certain error between the curvature value deduced by the compensation model and the actual value, and the relative error can meet the needs of practical applications. The main reasons for the error are that the theoretical analysis does not take into account the strain transfer efficiency between FBG and the sensor substrate, and there may be errors in the end face marking angle and the specific curvature arc of the site marking during the experiment.

## 5. Feasibility of High-Precision Monitoring of Straightness of Scraper Conveyor

**Figure 10:**
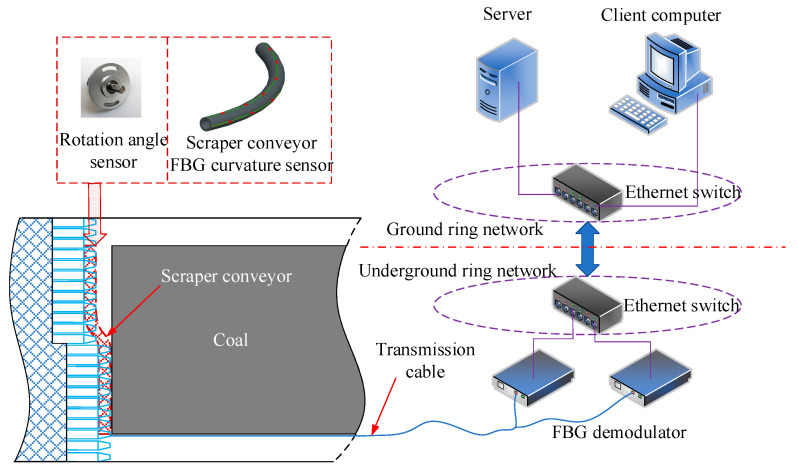
Schematic diagram of high-precision perception of straightness of scraper conveyor.

With the advancement of the working face, the scraper conveyor presents dynamic bending changes. As shown in Figure 10, the FBG wavelength variable caused by the curvature change of the scraper conveyor is obtained by fixing the FBG curvature sensor [22] along the scraper conveyor. The rotation angle sensor [29] is installed at the end of the sensor to obtain the rotation error angle of the sensor, and then the wavelength and other information are transmitted to the FBG demodulator by the transmission optical cable. The FBG demodulator converts the received optical signal into an electrical signal, and then it is transmitted to the server and the client computer of the ground dispatching command center through the underground ring network and the ground ring network. Based on the obtained wavelength data and angle information, the curvature information of high-precision discrete points of the scraper conveyor can be obtained through the accuracy compensation model based on the rotation error angle proposed in this paper, and then the high-precision three-dimensional shape of the scraper conveyor can be reconstructed through the three-dimensional curve reconstruction algorithm based on discrete points [30], so as to provide a data basis and guidance for the intelligent adaptive alignment of the scraper conveyor in the intelligent working face.

## 6. Conclusions

(1) Based on the development of an FBG curvature sensor of a scraper conveyor and combined with field work, we conclude that the rotation error angle is one of the main reasons for low sensing accuracy. On this basis, we constructed an accuracy compensation model based on the rotation error angle, and the formula for restoring the theoretical curvature of the sensor through the FBG wavelength change and the rotation error angle was derived theoretically.

(2) The strain characteristics of an FBG curvature sensor under different rotation error angles were analyzed by ANSYS Workbench numerical simulation software. Through the numerical simulation, the curvature radius was calculated according to the accuracy compensation model based on the rotation error angle constructed in this paper. The relative error of each discrete point is within ±0.9%, and the relative error after fitting is less than 0.2%. This verifies the effectiveness and rationality of the accuracy compensation model based on the rotation error.

(3) The experiment shows that within the rotation error angle range of 0~90°, the relative error of the curvature radius after fitting according to the theoretical formula is within ±3%, and the relative error of the curvature radius value deduced from each discrete point is within ±6%, which further confirms the correctness of the accuracy compensation model and can also meet the requirements for straightness perception accuracy of the scraper conveyor.

(4) Due to the workload, we did not develop a more applicable rotation error angle measurement instrument, and it is complex to use the scraper conveyor FBG curvature sensor together with the rotation angle sensor mentioned in Section 5. The influence of torsion on long-distance curvature detection cannot be ignored. This paper only details the influence of rotation error angle, and subsequent research on accuracy compensation based on torsion angle should be conducted on this basis. The temperature compensation design is not included in this paper. When the working face is under unfavorable conditions and the temperature cannot be kept constant, the temperature compensation design should be carried out on this basis.

## Figures and Tables

**Table 1:** Comparison between simulated and theoretical values of FBG under different rotation angle errors.

θ/°	Theoretical Strain of FBG I/με	Simulated Strain of FBG I/με	Relative Error of Strain	ρ/mm	Relative Error of ρ	Theoretical Strain of FBG II/με	Simulated Strain of FBG II/με	Relative Error of Strain	ρ/mm	Relative Error of ρ
0	2400.000	2397.800	0.092%	15,013.763	0.092%	0	5.781	-	-	-
30	2079.129	2089.300	−0.489%	14,927.005	−0.487%	−1201.442	−1194.400	−0.586%	−1194.400	0.829%
45	1698.250	1701.000	−0.162%	14,975.767	−0.162%	−1698.250	−1694.900	−0.197%	−1694.900	0.537%
60	1201.442	1203.200	−0.146%	14,978.106	−0.146%	−2079.129	−2085.900	0.326%	−2085.900	0.091%
90	0	5.781	-	-	-	−2400.000	−2397.700	−0.096%	−2397.700	0.576%

**Table 2:** Initial center wavelength and curvature sensitivity coefficient of FBG curvature sensor of scraper conveyor after packaging.

Grating Number	1	2	3	4	5	6
*λ_B_* of FBG I/nm	1529.750	1533.472	1537.890	1541.455	1545.551	1550.015
*M* of FBG I/nm·mm	42,955.380	43,059.894	43,183.951	43,284.056	43,399.072	43,524.421
*λ_B_* of FBG II/nm	1529.908	1533.737	1537.894	1541.481	1545.741	1550.033
*M* of FBG II/nm·mm	42,959.817	43,067.335	43,184.064	43,284.786	43,404.407	43,524.927

**Table 3:** Curvature obtained by fitting and its relative error.

Grating Number	FBG I	Relative Error	FBG II	Relative Error
1	14,973.217	−0.18%	15,289.569	1.93%
2	15,216.084	1.44%	14,987.86	−0.08%
3	15,046.869	0.31%	14,888.227	−0.75%
4	14,781.369	−1.46%	15,301.609	2.01%
5	15,051.658	0.34%	14,606.731	−2.62%
6	15,308.676	2.06%	14,921.489	−0.52%

**Table 4:** Variation of FBG central wavelength measured by experiment and calculated curvature.

FBG I-1 ^1^Δ*λ*	Calculated Value of *ρ*	Relative Error	FBG I-2Δ*λ*	Calculated Value of *ρ*	Relative Error	FBG I-3Δ*λ*	Calculated Value of *ρ*	Relative Error
2.840	15,125.542	0.84%	2.883	14,935.776	−0.43%	2.871	15,043.627	0.29%
2.478	15,019.519	0.13%	2.380	15,673.764	4.49%	2.416	15,482.917	3.22%
2.137	14,221.253	−5.19%	2.018	15,099.588	0.66%	2.153	14,193.017	−5.38%
1.355	15,873.646	5.82%	1.413	15,259.099	1.73%	1.384	15,620.999	4.14%
0.019	-	-	0.026	-	-	0.020	-	-
**FBG I-4Δ*λ***	**Calculated value of *ρ***	**Relative error**	**FBG I-5Δ*λ***	**Calculated value of *ρ***	**Relative error**	**FBG I-6Δ*λ***	**Calculated value of *ρ***	**Relative error**
2.878	15,042.118	0.28%	2.946	14,731.880	−1.79%	2.806	15,511.89	3.41%
2.601	14,419.104	−3.87%	2.458	15,295.102	1.97%	2.482	15,192.89	1.29%
2.064	14,838.377	−1.08%	2.004	15,323.095	2.15%	1.962	15,696.45	4.64%
1.468	14,758.260	−1.61%	1.438	15,104.094	0.69%	1.535	14,192.08	−5.39%
0.020	-	-	0.032	-	-	0.032	-	-
**FBG II-1Δ*λ***	**Calculated value of *ρ***	**Relative error**	**FBG II-2Δ*λ***	**Calculated value of *ρ***	**Relative error**	**FBG II-3Δ*λ***	**Calculated value of *ρ***	**Relative error**
0.036	-	-	0.031	-	-	0.021	-	-
−1.478	14,550.933	−0.030	−1.400	15,403.089	0.027	−1.447	14,941.797	−0.004
−1.966	15,461.999	0.031	−1.938	15,727.705	0.049	−1.922	15,899.304	0.060
−2.444	15,226.808	0.015	−2.402	15,529.388	0.035	−2.501	14,956.640	−0.003
−2.781	15,447.992	0.030	−3.036	14,185.739	−0.054	−3.006	14,367.816	−0.042
**FBG II-4Δ*λ***	**Calculated value of *ρ***	**Relative error**	**FBG II-5Δ*λ***	**Calculated value of *ρ***	**Relative error**	**FBG II-6Δ*λ***	**Calculated value of *ρ***	**Relative error**
0.022	-	-	0.027	-	-	−0.020	-	-
−1.400	15,480.896	0.032	−1.438	15,111.693	0.007	−1.503	14,498.959	−0.033
−1.940	15,783.937	0.052	−2.144	14,324.387	−0.045	−2.003	15,378.041	0.025
−2.439	15,373.458	0.025	−2.468	15,232.991	0.016	−2.575	14,642.833	−0.024
−2.890	14,976.038	−0.002	−3.059	14,189.764	−0.054	−2.897	15,022.534	0.002

^1^ FBG I-1 in the table represents the first grating measuring point of FBG I.

## Data Availability

All data and code used or analyzed in this study are available from the corresponding author on reasonable request.
